# Cohort profile: A prospective cohort study on newlywed couples in rural and poor urban Bangladesh

**DOI:** 10.1371/journal.pone.0316230

**Published:** 2025-01-22

**Authors:** Shaki Aktar, Shakil Ahmed, Tanjeena Tahrin Islam, Tarana -E-Ferdous, Meftah Uddin Ahmed, Syed Hassan Imtiaz, Salma Akter, Shathil Miah, Md. Sharful Islam Khan, Ruchira Tabassum Naved, Shams El Arifeen, Quamrun Nahar, Anisuddin Ahmed, S. M. Manzoor Ahmed Hanifi, Fauzia Akhter Huda

**Affiliations:** 1 Maternal and Child Health Division, icddr,b, Dhaka, Bangladesh; 2 Department of Health Research Methods, Evidence and Impact, McMaster University, Hamilton, Ontario, Canada; 3 Department of Data Science, RMIT University, Melbourne, VIC, Australia; 4 Health Systems and Population Studies Division, icddr,b, Dhaka, Bangladesh; IFPRI: International Food Policy Research Institute, UNITED STATES OF AMERICA

## Abstract

Sexual and Reproductive Health and Rights (SRHR) aim to enhance quality of life through safe sexual experiences, reproductive autonomy, and protection against gender-based violence. However, existing SRHR research and interventions in low- and middle-income countries like Bangladesh predominantly focus on women, often understating men and neglecting the nuanced contextual issues faced by married couples. This study contributes to filling this gap by examining SRHR dynamics among newlyweds in rural and poor urban areas of Bangladesh, especially focusing on marital satisfaction, fertility preferences, and post-marriage adaptation mechanisms. Employing a prospective cohort design across four Health and Demographic Surveillance Systems (HDSS) managed by icddr,b, the study spans from November 2021 to March 2025, with data collection starting in December 2022. Of the 2011 newlywed couples identified, 666 who met eligibility criteria (married for ≤6 months, first marriage, and no pregnancy history) were enrolled. Participants will undergo six quantitative interview sessions over a two-year period. Additionally, 44 in-depth qualitative interviews were conducted with 22 purposefully selected couples. Demographic data reveal that a significant proportion of husbands (67.3% in rural areas, 71.8% in poor urban areas) are aged 20–29 years, while a majority of wives (67.9% in rural areas, 84.8% in poor urban areas) are adolescents. Education levels varied, with a higher proportion of poor urban husbands lack formal education compared to their rural counterparts (7.2% vs. 3.0%), while no significant variation was observed among wives (0.6% vs 1.0%). Arranged marriages are more common among rural couples (80%) compared to those in poor urban areas (50%). Moreover, poor urban participants tend to marry at a younger age than the rural participants, with poor urban wives marrying earlier than rural wives (60.4% vs 39.7%). This pioneering study provides valuable insights into the SRHR needs of newlywed couples in Bangladesh. The findings will be instrumental for designing targeted interventions aimed at improving SRHR service utilization and enhancing overall well-being, particularly in rural and poor urban areas of the country.

## Background

Sexual and reproductive health and rights (SRHR) are essential for human dignity and well-being, encompassing the rights to safe and fulfilling sexual experiences, autonomy in reproductive decisions, access to supportive services, and protection and prevention against gender-based violence (GBV) [1]. Despite significant progress in improving SRHR information and services in Bangladesh over the past two decades, inequalities remain across diverse population groups and regions, complicating efforts to achieve Sustainable Development Goals (SDGs) related to SRHR [2].

Marriage is often viewed as a key institution for family life and future generations, offering personal development, financial security, and overall well-being. However, these benefits can be undermined by harmful practices, such as early marriage, dowry disputes, and violence against women (VAW), posing significant risks to sexual and reproductive health, particularly for women. These issues remain prevalent in Bangladesh and other low- and middle-income countries (LMICs), creating substantial barriers to SRHR progress [3–7].

Newlywed couples, often overlooked in SRHR initiatives, hold the potential for significant impact on attaining SRHR if their specific needs are addressed [8–11]. Adolescent and young girls in Bangladesh, who often marry early and face numerous SRHR challenges–including limited knowledge and understanding of sexuality, family planning, and reproductive health–make up a significant portion of the population [12]. The lack of adequate SRH education in schools and the prevailing social stigma surrounding SRH information exacerbate this issue [13–15]. Conversely, couples’ health and well-being are influenced by informed decision-making regarding fertility preferences and healthcare-seeking behaviours, which are affected by access to family planning services, education, economic conditions, and socio-cultural norms [16–18]. Moreover, the transition from singlehood to marriage presents challenges, particularly for young couples adjusting to new environments and relationships.

Sexual activity within marriage plays a crucial role in marital satisfaction and overall well-being, yet experiences of sexual fulfilment can differ due to gender dynamics and power imbalances. Societal taboos surrounding sexuality often prevent open discussions about sexual health, particularly for women, resulting in unmet sexual needs and barriers to seeking care [19]. This lack of communication can lead to emotional distress, disproportionately affecting women, and may contribute to issues such as domestic violence and divorce [20,21]. A 2022 survey by the Bangladesh Bureau of Statistics (BBS) revealed that divorce rates in the country have doubled, increasing from 0.7% in 2021 to 1.4% in 2022 [22].

In recent years, intimate partner violence (IPV) has emerged as a growing concern in Bangladesh, reflecting trends seen in other LMICs. Bangladesh records one of the highest rates of lifetime IPV among ever-married women, with estimates ranging from 55% to 95%, while the prevalence among men remains below 1% [23–26]. This disparity is largely due to societal and cultural practices, as well as the male dominance in Bangladeshi society. Newlywed women are particularly vulnerable to IPV, often due to dowry disputes, early marriages, and spousal age differences [24].

Furthermore, the vulnerability of married women to sexual exploitation in the absence of their husbands—often due to labour migration—is a significant concern in Bangladesh [27]. Marrying before migrating to work overseas is a common practice among migrant workers, leaving wives to reside with their in-laws [28]. This situation exposes women to social disadvantages and stigma, occasionally resulting in psychological, physical, and sexual abuse from members of their husbands’ families, neighbours, relatives, and others, while also heightening the risks of reproductive tract infections (RTIs), sexually transmitted infections (STIs), and social disharmony [27].

**Fig 1:**
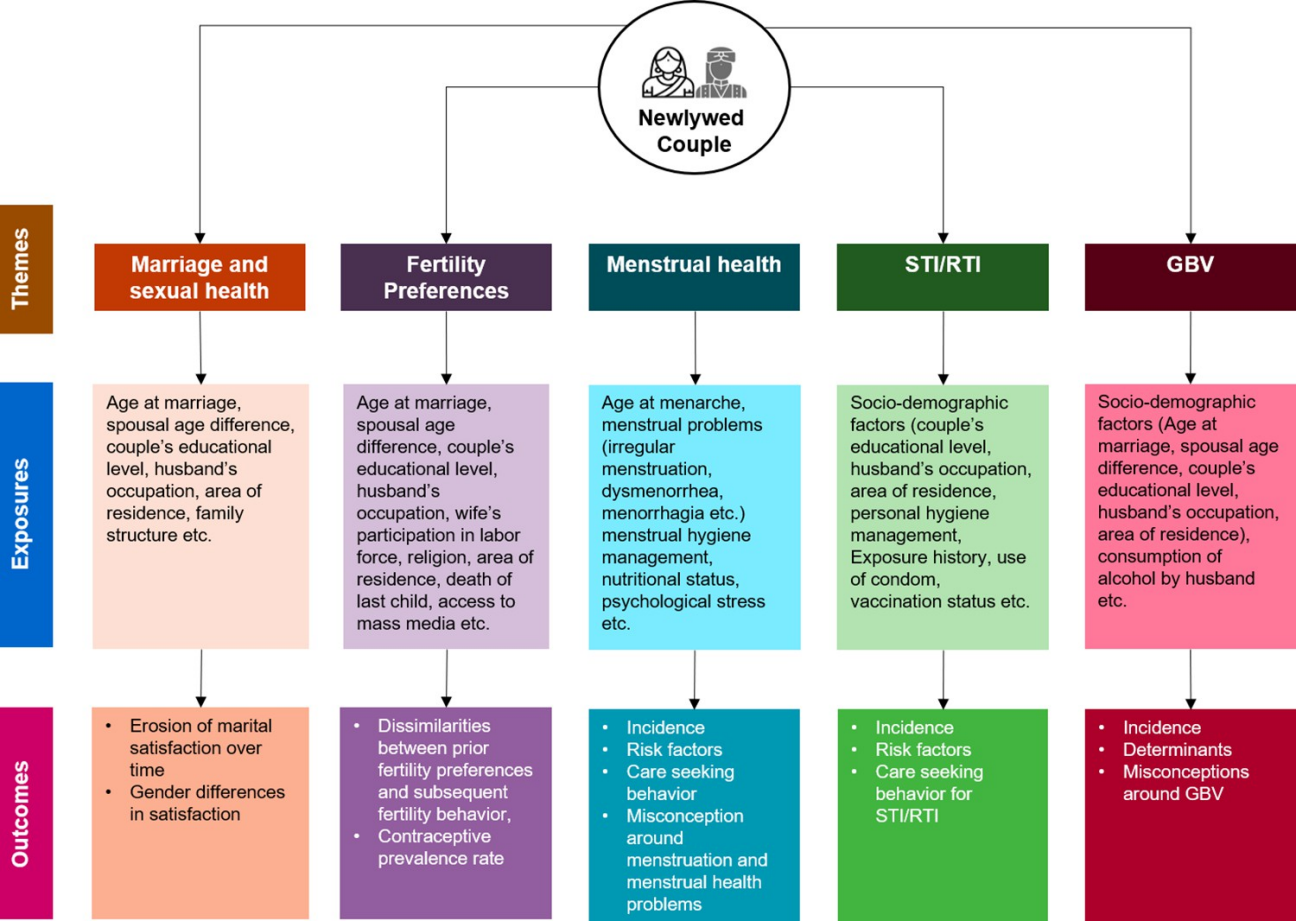
Conceptual framework for understanding the SRHR needs of newlywed couples in Bangladesh.

Despite its critical importance, research on the complex psychological, social, and cultural dynamics affecting newlywed couples, particularly in Bangladesh and similar LMICs, remains scarce [29–33]. Therefore, this study aims to understand the contexts, needs, care-seeking behaviours, barriers, and challenges around SRHR among newlywed couples in selected rural and poor urban areas of Bangladesh. To achieve this larger goal, the study will focus on the following specific objectives: i) Establish a cohort of newlywed couples; ii) Measure changes in marital satisfaction over time among newlywed couples; iii) Identify factors influencing fertility preferences, contraceptive method choice and use among newlywed couples; v) Estimate the incidence, risk factors, and care seeking behaviours for STI/RTI among newlywed couples, and for menstrual problems among newlywed females; vi) Measure the incidence and determinants of intimate partner violence (IPV) among newlywed females; and vii) Explore the coping strategies adopted by newlywed couples after marriage. The conceptual framework of the study is illustrated in Fig 1.

In this paper, we will detail the methodology used to form the cohort and provide an overview of their sociodemographic profile, with particular emphasis on their marriage dynamics.

## Methods

### Study design

This is a prospective cohort study utilizing both quantitative and qualitative methods for data collection. We have established a cohort of newlywed couples to track their life events, with a particular focus on marital satisfaction over time, sexual and reproductive health, fertility preferences, contraceptive method choice and use, RTIs/STIs, intimate partner violence (IPV), and sexual vulnerabilities. Additionally, we aim to explore couples’ experiences with adjustment strategies and changes in marital relationships over time, decision-making processes regarding fertility and contraceptive choices, as well as existing beliefs and misconceptions surrounding menstruation and IPV through qualitative methods.

#### Study duration

The study extends over 41 months, from November 2021 to March 2025. This timeframe includes activities such as developing research protocol and data collection tools, pre-testing these tools, obtaining IRB approval, creating tablet-based programs, recruiting and training staff, conducting rounds of quantitative data collection, carrying out and transcribing in-depth interviews (IDIs), data cleaning and processing, coding, analysis, report writing, and dissemination of findings.

### Study areas

We are conducting this study across four Health and Demographic Surveillance Systems (HDSS) areas managed by icddr,b, including two rural areas (Matlab and Chakaria) and two poor urban areas (Korail and Mirpur slums). icddr,b has a history of systematically collecting sociodemographic and health-related data through these HDSS areas, including information on births, deaths, migrations, marriages, divorces, education, employment, household structure, family planning, maternal health, and pregnancy outcomes (livebirths, stillbirths, induced and spontaneous miscarriages).

***Matlab HDSS*:** Located in Matlab, Chandpur district, Bangladesh, approximately 75 km southeast of Dhaka city, the Matlab HDSS has been collecting surveillance since 1966. It covers over 220,000 residents across 142 villages. The site is divided into two zones based on health service accessibility: the icddr,b service area and the government service area. Our study operates within the government service area, where healthcare is administered through public health facilities without icddr,b’s direct service provision. Surveillance data is collected every three months by icddr,b’s Community Health Research Workers (CHRWs).

***Chakaria HDSS*:** Established in 1999, this surveillance site is located in Chakaria, Cox’s Bazar district, on the south-eastern coast of the Bay of Bengal. It covers approximately 19,200 households with a population of around 92,000, distributed across 49 villages. Data collection occurs every three months to support local health initiatives targeting children, women, and vulnerable populations.

***Poor urban HDSS*:** This HDSS includes selected slum areas in Gazipur, Dhaka North, and Dhaka South City Corporations. Since 2015, icddr,b has been overseeing health-related services in these areas through the Urban Primary Health Care Project (UPHCP), targeting sizable slum communities with at least 150,663 households. Community health workers conduct household visits every three months to update surveillance data. The Korail and Mirpur HDSS areas are located within the Dhaka North City Corporation.

### Cohort formation

To build the cohort, our sampling frame includes only newlywed couples. To be in the sampling frame, couples must have been residents of the study areas for at least six months and hold unique identification numbers provided by the respective HDSS. We have defined newlywed couples as those married for six months or less at the time of enrolment [34]. Only couples who have been married once are included. They have all provided written informed consent to participate. Couples where either spouse did not provide consent were excluded from the study. Moreover, we excluded couples who were pregnant or experienced a miscarriage within six months of marriage at the time of enrolment. The inclusion and exclusion criteria for the study are outlined in Table 1.

**Table 1:** Inclusion and exclusion criteria for the study.

Inclusion criteria	Exclusion criteria
• Residents of the study areas for at least the last six months. • Each possesses a unique identification number provided by the respective HDSS. • First marriage for both spouses. • Married for six months or less. • Provided written informed consent to participate.	• Currently pregnant. • Any occurrences of miscarriage within six months of marriage.

### Enrolment procedures

***Identification of newlywed couples*:** Initially, we extracted lists of newlywed couples from the databases of each surveillance site, namely Matlab, Chakaria, Mirpur, and Korail. Subsequently, data collectors from the study team visited each residence to verify the accuracy of the lists provided by the HDSS and ensure that the couples met the study’s inclusion and exclusion criteria.

***Enrolment of study participants*:** Before enrolment, we provided detailed information to eligible couples about the study’s objectives, procedures, potential risks, benefits, follow-up visit schedules, and other pertinent details. Moreover, newlywed wives had to meet specific criteria: those experiencing regular menstruation and reporting a missed period of 14 days or more, or those suspected of pregnancy, were offered a pregnancy test using a pregnancy strip. These tests were conducted with due privacy. Couples with a positive pregnancy test were excluded from the study. Enrolment proceeded only after both spouses provided written informed consent.

Enrolment continued until the required sample size was achieved. Throughout the enrolment process, the study’s data collection team was accompanied by surveillance workers during routine household visits. The enrolment procedure was standardized across all four study areas and conducted simultaneously until the required sample size was met. The enrolment periods were: Matlab: 19/12/2022 to 30/05/2023; Chakaria: 21/12/2022 to 29/04/2023; Mirpur: 16/12/2022 to 16/06/2023; and Korail: 16/12/2022 to 29/05/2023. This paper presents only the enrolment data from these periods and does not include follow-up data.

**Fig 2:**
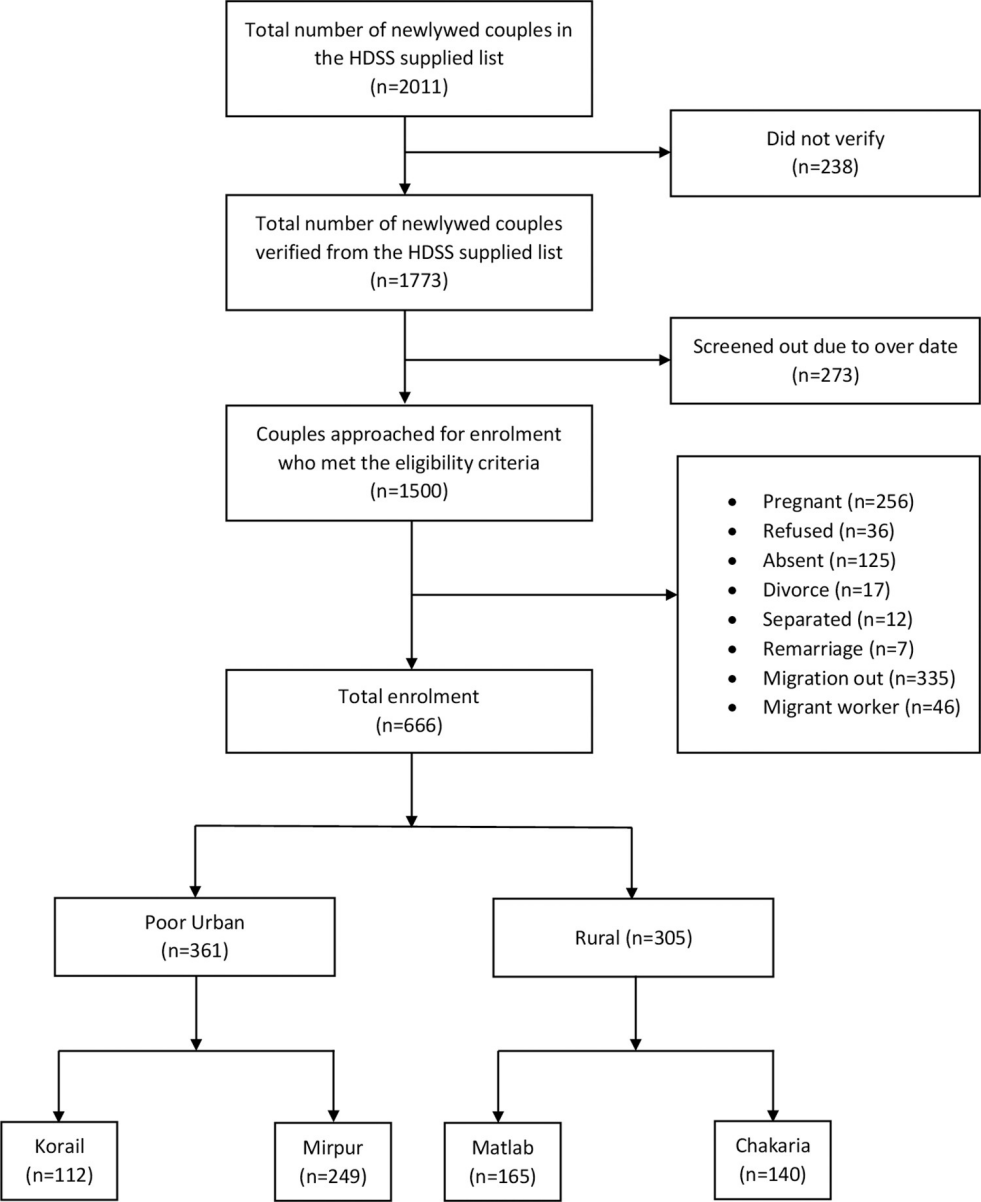
Flowchart of study population selection.

The study team received lists of 2,011 newlywed couples from all HDSSs during the enrollment period. Of these, 1,773 couples were verified at the household level. Out of this pool, 273 couples were excluded for exceeding the six-month mark. Approximately 1,500 newlywed couples who met the eligibility criteria were approached for enrollment. Exclusions were made for pregnant individuals (n = 256), refusals (n = 36), absences (n = 125), migration (n = 335), divorces (n = 17), separations (n = 12), remarriages (n = 7), and husbands who were migrant workers at the time of enrollment (n = 46). Finally, 666 newlywed couples were enrolled and included in the study. Among them, 305 couples were enrolled in the two rural areas (165 in Matlab and 140 in Chakaria), and 361 couples were enrolled in the two poor urban areas (249 in Mirpur and 112 in Korail slums), based on the population size of the study areas. The enrollment numbers are illustrated in the flow chart in Fig 2.

#### Follow-up of study participants

Following enrolment, we began regular follow-up with the newlywed couples. The data collection team conducts visits every four months, resulting in a total of six rounds of data collection over 24 months, including the initial enrolment visit. The follow-up data collection process is ongoing. To date, we have completed three follow-up visits, and are preparing for the final two rounds.

We have predefined criteria for handling cases of pregnancy, divorce, separation, migration, or loss of communication. If a couple becomes pregnant, we will continue to follow up with them to understand their knowledge, attitude, practices, care seeking behaviour, and experiences related to pregnancy and childbirth. Couples who remain separated without formal divorce, will also be monitored. Pregnant, migrant, and divorced couples were excluded only at the time of recruitment, as our target was to enrol newlywed couples who were currently married and had no prior pregnancy experience. During follow-up visits, all couples—regardless of pregnancy status or whether one spouse has migrated for work—will be approached for data collection.

We are also following up with couples who have relocated outside the study area but remain within the vicinity. For migrant spouses living outside Bangladesh, we are collecting data through phone interviews. Couples who have separated but have not yet divorced will continue to be included in the study, allowing us to gain insights into the reasons for their separation. Hence, we will continue collecting data from separated couples until formal divorce is finalized.

**Fig 3:**
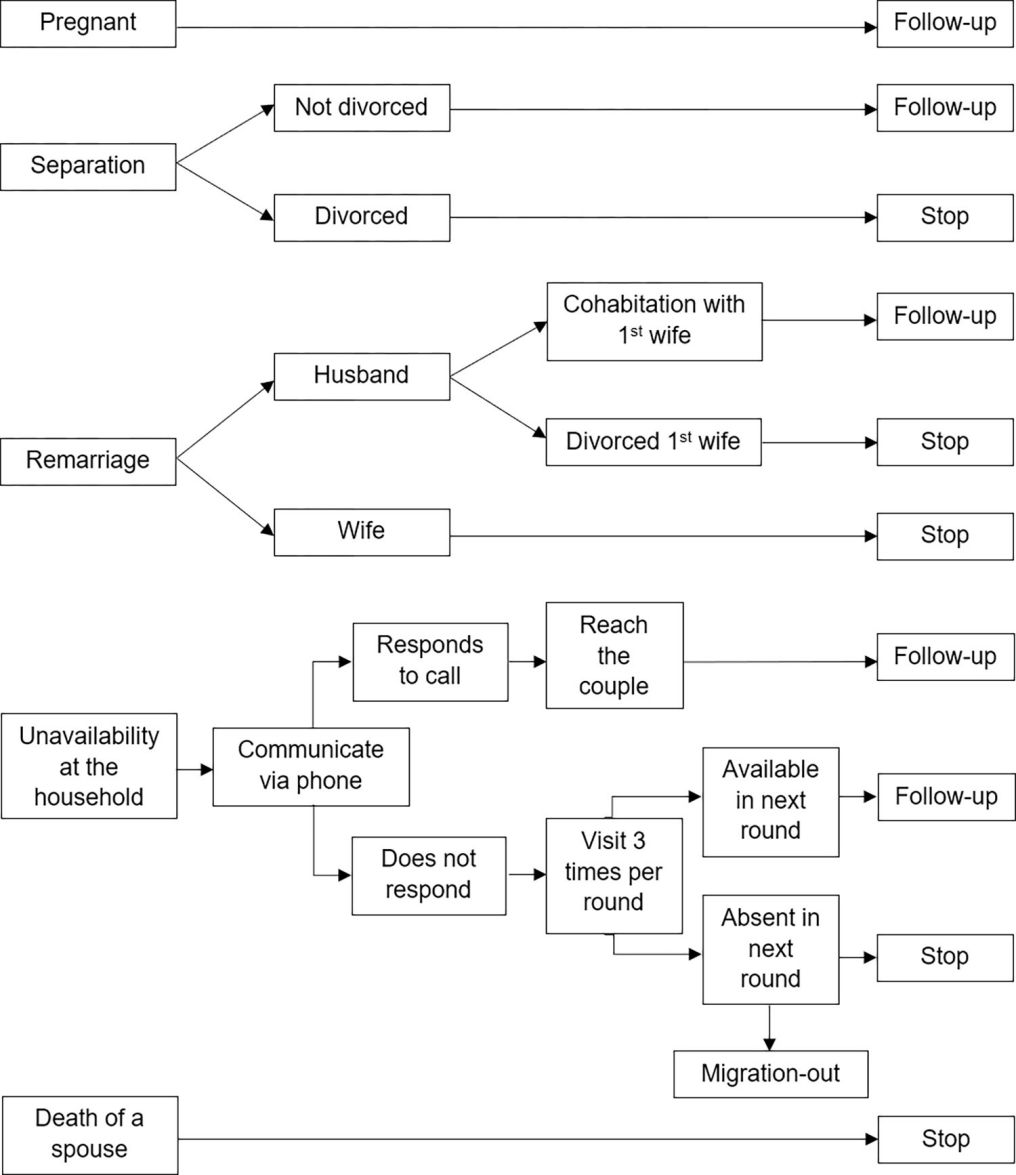
Follow-up decision mechanism.

In case of remarriage, if a male partner marries another person but continues to live with the enrolled partner, we will maintain follow-up with the couple. However, in the Bangladeshi context, a woman must formally divorce her partner before remarrying. Therefore, if the enrolled wife remarries, the couple will be classified as dropouts. Fig 3 depicts the follow-up decision mechanism.

If a couple is unavailable during visits or moves out of an HDSS area, we will attempt to reach them by phone. If contact is established, we will arrange follow-up visits. For couples who are neither present at their current address or reachable by phone, we will make at least three attempts per follow-up round. If they are unreachable after three consecutive visits, they will be marked as “absent”, and follow-up visits will be discontinued. Follow-up will also cease in the event of a participant’s death.

### Data collection procedure

#### Quantitative data collection

The quantitative data collection process involved several preparatory stages. We adapted most of the tools from standardized instruments used in the Bangladesh Demographic and Health Survey [5] and other longitudinal studies.

Initially, we pre-tested the data collection tools in both rural and poor urban settings to refine them. Simultaneously, we developed and improved a tablet-based data collection system based on feedback from these pre-tests. We now use the refined toolset within the tablet-based system to collect quantitative data.

***Baseline information*:** During enrolment, we collected baseline data on couples’ sociodemographic characteristics, marriage and sexual health, menstruation and menstrual health-related problems (for wives only), fertility preferences, STIs/RTIs, intimate partner violence (IPV), and sexual vulnerability. We classified early marriages as those where husbands married before age 21 and wives before age 18. We also inquired about the type of marriage–arranged or autonomous. Arranged marriages are those initiated by family members, while autonomous marriages are initiated by the couples themselves. Marriages involving family involvement are categorized as the hybrid model (love-cum-arranged), and those without family involvement are considered the western model [35].

***Anthropometry*:** We recorded anthropometric measurements (height, weight, and mid-upper arm circumference), temperature, and blood pressure of the wives. To ensure gender sensitivity, female interviewers conducted interviews with wives and male interviewers with husbands. All information, except sociodemographic details, including anthropometric and biometric measurements, is collected repeatedly at subsequent visits to observe trends over time.

***Marital satisfaction*:** We used the 15-item ENRICH Marital Satisfaction (EMS) scale to evaluate relationship issues, communication, and happiness among newlywed couples. The scale includes 10 items on marital quality (Marital Satisfaction Scale) and 5 items on positive marital descriptions (Idealistic Distortion Scale) [36].

***Intimate partner violence (IPV)*:** We employed an 11-item tool to assess IPV against both rural and poor urban wives. This tool covers physical and sexual violence, with six items focusing on physical assault and five on sexual assault. A positive response to any item in either domain classified the wife as a victim of IPV [37].

Both the core researchers and interviewers received training on “Mainstreaming Gender and Rights in Public Health Research” prior to the commencement of the study. The training manual was largely based on the WHO’s training manual for health managers. All interviews are conducted in private and with a non-judgmental approach. Interviewers reassure respondents of the confidentiality of the information they provide. During interviews, a separate room is used, and the topic switched if any family members, relatives, or neighbours unexpectedly arrive. Additionally, a dummy questionnaire on a separate topic is carried to demonstrate to husbands, in-laws, or others who may be curious about the subject matter. If the study team identifies any form of violence and the respondent seeks help, the interviewer provides information about nearby victim support centres.

***Sexual vulnerabilities*:** A 5-item Sexual Harassment Scale (SHS) assessed the sexual vulnerability of wives [38]. Each item offered “Yes” or “No” responses, with any affirmative response categorizing the wife as sexually vulnerable.

***Pregnancy and childbirth*:** After enrolment, a pregnancy test utilizing pregnancy strips is offered at each follow-up visit to wives who report a missed period lasting 14 days or more or are suspected of being pregnant. If the test is positive, we follow up with the couples to gather details about the pregnancy and delivery, including antenatal care (ANC), miscarriages, delivery, postnatal care (PNC), any complications, and their management.

Enrolment and follow-up visit schedules, as well as the information to be collected, are detailed in Table 2.

**Table 2:** Enrolment and follow-up visit schedule and data collection.

Visit Number	Time Frame	Information Collected
Enrolment visit (Visit 1)	Within 6 months of marriage	• For both spouses: sociodemographic characteristics, marriage and sexual health, fertility preferences, and STIs/RTIs. • For wives specifically: menstrual health, IPV, sexual vulnerability, anthropometric measurements (height, weight, mid-upper arm circumference), temperature, and blood pressure.
Follow-up 1 (Visit 2)	4 months after enrolment	• Sociodemographic data excluded. • A repeat collection of all information gathered during Visit 1. • Collection of pregnancy and ANC information.
Follow-up 2, 3, 4, 5 (Visits 3, 4, 5, 6)	At intervals of 8, 12, 16, and 20 months after enrolment, respectively.	• A repeat collection of all information gathered during Visit 2. • Collection of delivery and PNC information.

#### Qualitative data collection

We have already conducted 44 in-depth interviews (IDIs) with both spouses of 22 couples. The final number of IDIs will be determined by data saturation. To reduce the potential for information bias, interviews with each couple are conducted simultaneously. However, to mitigate the risk of retaliation from either spouse, husbands and wives are interviewed in separate rooms. To ensure confidentiality, privacy and a neutral, comfortable environment, the IDIs are conducted in our study office, which also provides an uninterrupted setting for the interviews. Participants are informed about our confidentiality and privacy policies and are asked not to share the content of the interviews with their spouse. Gender sensitivity is maintained by assigning male interviewers to husbands and female interviewers to wives.

Through these qualitative interviews, we aim to explore selected aspects of the couple’s lives, including adjustment strategies after marriage, changes in marital relationships over time, decision-making processes related to fertility preferences and contraceptive method usage, as well as perceptions and beliefs surrounding menstruation and intimate partner violence.

Initially, IDI guidelines were developed based on study objectives, pre-tested in both poor urban and rural areas, and refined based on feedback. Guidelines for husbands and wives were mostly similar in structure except for gender-specific issues such as IPV and pregnancy, for which the IDI was slightly detailed. IDI participants were selected based on a wide range of criteria, such as type of marriage, spousal age gap, education, occupation, socio-economic status, and IPV.

Each IDI commenced upon written informed consent from the participant. We conducted all IDIs in Bangla, with site-specific data collectors acting as interpreters for clarifying local dialects. IDIs were audio-recorded using a tape recorder, with interviewers taking field notes. IDIs are being transcribed verbatim and enriched with field notes, and the audio records will be destroyed after transcription. Transcriptions are being anonymized to ensure confidentiality.

#### Ethical approval

This study was approved by the Institutional Review Board (IRB) of icddr,b. (PR#22095). All participants provided informed written consent to join the study. Since our target population consisted of married couples, even minor participants could give their own consent due to their marital status, so parental or guardian consent was not required.

#### Sample size calculation

*Quantitative*. In each study site, a target of 500 respondents, or 250 couples, was set to be followed-up throughout the study period. To attain this number, the calculations accounted for a 20% out-migration rate for the poor urban site, 6% for the rural site, and 10% non-response. Based on these factors, an estimated 714 respondents (or 357 = ~ 360 couples) were needed from the urban site and 596 respondents (or 298 = ~ 300 couples) from the rural site, totalling 660 couples for enrolment.

The study aims to estimate different proportions with varying denominators to better understand the context related to sexual and reproductive health and rights (SRHR) among newlywed couples. Since no prior data is available on the SRHR needs of newlywed couples, the sample size was calculated to provide adequate power to estimate different prevalence rates with acceptable precision, using 95% confidence intervals (Table 3A).

**Table 3:** a. Sample size calculation. b. Sample size calculation.

Prevalence		Precision	
0.01		0.012	
0.02		0.017	
0.05		0.027	
0.1		0.037	
0.15		0.044	
0.2		0.05	
0.25		0.054	
0.3		0.057	
0.5		0.062	
0.6		0.061	
0.7		0.057	
0.8		0.05	
0.9		0.037	
Prop. (p1)	Prop. (p2)		Estimated minimum power
0.01	0.06		86.3%
0.02	0.08		87.1%
0.05	0.12		80.3%
0.1	0.19		81.7%
0.15	0.25		80.0%
0.2	0.31		80.8%
0.25	0.37		82.9%
0.3	0.42		80.0%
0.5	0.63		83.7%
0.6	0.72		81.0%
0.7	0.81		81.8%
0.8	0.9		88.2%
0.9	0.97		89.0%

The adequacy of the sample size for testing the difference between two proportions (p1 vs p2) for changes in menstrual problems, experiences of intimate partner violence, and other factors, with a minimum of 80% power and 95% confidence intervals, is shown in Table 3B.

*Qualitative*. We are following standard practice in qualitative research until data saturation is reached. Our research questions focus on the social norms experienced by newlywed couples. Hence, primarily we assume that approximately 10–12 couples from each study site will provide the necessary information relevant to our study aims.

We conduct the interviews following the IDI guidelines, ensuring a natural flow of conversation. We review each set of IDIs on the day of the interview to identify key findings. This review process helps us to decide if data saturation has been reached, meaning that no new information is emerging on the selected topics. We will declare completion of qualitative data collection based on achieving data saturation across the research sub-topics. Thus, final number of IDIs may change based on data saturation.

#### Data analysis plan

*Quantitative*. We aim to understand how newlywed couples from the selected poor urban and rural areas progress in their marital lives in terms of marital satisfaction, decision-making, contraceptive use, family planning, and coping mechanism. Therefore, we will analyse trends of these topics of interest through the six rounds of data. We will measure the contributing factors behind the topics of interest through logistic regression. We will conduct a comparative analysis between couples and individuals from poor urban and rural areas. The overall data analysis plan is provided in detail in Table 4.

**Table 4:** Data analysis plan.

Key outcome variables	Research questions	Control variables	Using tools
Measure changes in marital satisfaction over time among newlywed couples	1. How does marital satisfaction change over time among newlywed couples living in rural and poor-urban areas of Bangladesh?	1. Age at marriage 2. Spousal age difference 3. Wealth index 4. Couple’s education 5. Couple’s occupation 6. Type of marriage 7. Residence: Rural/poor-urban 8. IPV: Physical violence/sexual violence 9. Pregnancy history: Ever pregnant vs. not pregnant yet 10. Pregnancy outcomes: live birth (M/F) & still birth	1. Mean and standard deviation (over time) 2.General estimating equation (GEE) with 95% confidence intervals (CI) in final model
	2. What are the differences in marital satisfaction between husbands and wives in rural and poor-urban areas of Bangladesh?		
	3. What factors significantly impact marital satisfaction among newlywed couples in rural and poor-urban settings in Bangladesh, and how do these factors contribute to their overall satisfaction?		
Identify factors influencing fertility preferences, contraceptive method choice and use among newlywed couples	1. How do newlywed couples in rural and poor urban areas of Bangladesh make decisions about family planning, and what factors influence their choices?	1. Husbands staying abroad 2. Husbands/family members preferences 3. Age at marriage 4. Spousal age differences 5. Knowledge on family planning methods 6. Desire for children 7. Contraceptive method choice 8. Contraceptive method usage	1. Frequency distribution 2.General estimating equation (GEE) with 95% confidence intervals (CI) in final model
	2. How knowledgeable are newlywed couples in rural and poor-urban areas of Bangladesh about family planning and contraception, and what are their attitudes and practices regarding these methods?		
	3. What are the preferences and decision-making processes of newlywed couples in rural and poor-urban areas of Bangladesh regarding expanding their family?		
Estimate the incidence, risk factors, and care seeking behaviors for STI/RTI among newlywed couples, and for menstrual problems among newlywed females	1. What is the incidence of STI/ RTI among the newlywed couples?	1. Age at menarche 2. BMI 3. Use of sanitary pad 4. Residence: Rural/Urban 5. Desire for children 6. Couple’s education 7. Couple’s occupation 8. Use of condom 10. Reporting of menstrual problems 11. Care seeking for menstrual problems 12. Care seeking for STI/RTI problems	1. Frequency distribution 2.General estimating equation (GEE) with 95% confidence intervals (CI) in final model
	2. What are the risk factors for STI/ RTI among the newlywed couples in rural and poor-urban Bangladesh		
	3. What is their health-care seeking behaviour for STI/RTI?		
Measure the incidence and determinants of intimate partner violence (IPV) among newlywed females;	1. What factors contribute to the occurrence of IPV in the lives of newlywed women in rural and poor-urban areas of Bangladesh?	1. Dowry 2. Wealth index 3. Age at marriage 4. Spousal age difference 5. Couple’s education 6. Couple’s occupation 7. Type of marriage 8. Residence: Rural/poor-urban 9. IPV: Physical violence/sexual violence 10. Pregnancy history: Ever pregnant vs. not pregnant yet 11. Pregnancy outcomes: live birth (M/F) & Still birth	1. Frequency distribution 2.General estimating equation (GEE) with 95% confidence intervals (CI) in final model
	2. Do the frequencies and patters of IPV experienced by newlywed women in rural and poor-urban areas of Bangladesh change over time?		
	3. What measures do newlywed women in rural and poor urban areas of Bangladesh take to protect themselves against IPV?		

*Qualitative*. We will employ the inductive thematic analysis approach for analysing qualitative data. Before commencing the coding of the IDIs, we will develop an *a priori* codebook based on the guidelines. We will then systematically index or code the data using the *a priori* codes, while also adding new codes that emerge from the IDIs. Multiple researchers will code the same sections of text to assess coding reliability. Contradictory codes will be compared and discussed until the researchers come into a common consensus. Once coding of all IDIs is completed, we will identify patterns and associations among the codes to narrow down to sub-themes and overarching themes. After finalizing the themes, we will develop an analytical narrative describing each theme and sub-theme. This narrative will highlight both common and unusual issues that emerge. To support our findings, we will use relevant quotes (translated) from the IDIs.

## Findings

### Socio-demographic characteristics of enrolled newlywed couples

Among the enrolled newlywed couples, over two-thirds of husbands from both rural (67.3%) and poor urban (71.8%) areas were in the 20–29 age group. In contrast, three out of four (77.3%) wives were aged between 13 and 19 years (rural vs. poor urban: 67.9% vs. 84.8%) (Table 5).

**Table 5:** Sociodemographic information of enrolled newlywed couples.

Sociodemographic information		Rural		Poor urban	
		Husband (n = 305)	Wife (n = 305)	Husband (n = 361)	Wife (n = 361)
		n (%)	n (%)	n (%)	n (%)
**Age at enrolment** **(in years)**	13–19	21 (6.9)	207 (67.9)	85 (23.6)	306 (84.8)
	20–24	106 (34.8)	80 (26.2)	198 (54.9)	47 (13.0)
	25–29	99(32.5)	15 (4.9)	61 (16.9)	6 (1.7)
	≥30	79 (25.8)	3 (1.0)	17 (4.7)	2(0.6)
**Education**	No education	9 (3.0)	3 (1.0)	26 (7.2)	2 (0.6)
	Primary incomplete	61 (20.0)	8 (2.6)	96 (26.6)	65 (18.0)
	Primary complete^1^	23 (7.5)	7 (2.3)	59 (16.3)	47 (13.0)
	Secondary incomplete	89 (29.2)	140 (45.9)	104 (28.8)	174 (48.2)
	Secondary complete^2^ or higher	123 (40.3)	147 (48.2)	76 (21.1)	73 (20.2)
**Income generation in the last 12 months**	Yes	293 (96.1)	75 (24.6)	354 (98.1)	174 (48.2)
	No	12 (3.9)	230 (75.4)	7 (1.9)	187 (51.7)
**Religion**	Islam	275 (90.2)		358 (99.2)	
	Hinduism	30 (9.8)		3 (0.8)	
Type of family*	Nuclear	8 (2.6)		80 (22.2)	
	Joint	297 (97.4)		281 (77.8)	

^1^Primary complete: Grade V (five) completed

^2^Secondary complete: Grade X (ten) completed.

Overall, 3.0% of rural husbands and 7.2% of poor urban husbands had no formal education, compared to 1.0% of rural wives and 0.6% of poor urban wives. A higher proportion of wives in both areas had completed more than primary education compared to their husbands (94.1% vs. 69.5% in rural areas and 68.4% vs. 49.9% in poor urban areas). However, completion of secondary or higher education was more common among rural couples than among those from poor urban areas (Table 5).

Most (97.1%) husbands from both rural and poor urban areas were engaged in income-generating activities over the past 12 months. In contrast, one-fourth (24.6%) of rural wives and nearly half (48.2%) of poor urban wives were involved in income generation during the same period (Table 5).

Almost 94.7% of the total study population practiced Islam. There were more Hindu participants in the rural areas (9.8%) in compare to poor urban (0.8%) (Table 5).

Around 88% of the study participants lived in joint families. This figure was nearly 98% in rural regions, while about 22% of participants in urban areas lived in nuclear families (Table 5).

### Marriage dynamics and preferences

Wives typically preferred to marry after turning 18, with a higher average preferred age in rural areas. Husbands, on average, preferred marriage at 24 years, with rural husbands favouring it at 25 years—almost one year older than their urban counterparts. Consequently, urban participants favoured earlier marriages compared to those in rural areas (Table 6).

**Table 6:** Marriage dynamics of the cohort.

Characteristics		Rural		Poor urban	
		Husband (n = 305)	Wife (n = 305)	Husband (n = 361)	Wife (n = 361)
**Preferred age for marriage (in years)**	Mean	25.1	19.6	22.9	18.8
	Median	25.0	19.0	22.0	18.0
**Age at marriage** **(in years)**	Mean	25.9	18.7	22.2	17.0
	Median	26.0	18.0	21.0	17.0
		n (%)	n (%)	n (%)	n (%)
**Timing of marriage**	Early^1^	45 (14.8)	121 (39.7)	132 (36.6)	218 (60.4)
	Timely^2^	260 (85.2)	184 (60.3)	229 (63.4)	143 (39.6)
**Type of marriage**	Arranged	247 (80.5)		158 (43.8)	
	Autonomous	58 (19.5)		203 (56.2)	

The actual average age at marriage was 24.0 years for husbands and 17.8 years for wives. In rural areas, husbands married at an average age of about 26 years, which is four years older than the average age for poor urban husbands. For wives, the average age at marriage was 17 years in poor urban areas, which is 1.7 years younger than in rural areas (Table 6).

The proportion of early marriages among poor urban husbands was approximately twice that of rural husbands (36.6% vs 14.8%). Similarly, about 60.4% of poor urban wives and 39.6% of rural wives experienced early marriages. Overall, more than half (54.6%) of the wives were married before the age of 18 (Table 6).

In terms of marriage arrangements, over four-fifths of rural couples (80.5%) reported having arranged marriages, while the remainder had autonomous marriages (19.5%). Conversely, almost half of the couples in poor urban areas had autonomous marriages (56.2%) (Table 6).

#### Sociodemographic information of qualitative interview participants

To date, we have conducted IDIs with both spouses of 22 couples. These include 4 couples from Korail, 5 couples from Chakaria, 6 couples from Mirpur, and 7 couples from Matlab. The sociodemographic information of the IDI participants is shown in Table 7.

**Table 7:** Sociodemographic information of qualitative interview participants.

Sociodemographic information		Rural		Poor urban	
		Husband (n = 12)	Wife (n = 12)	Husband (n = 10)	Wife (n = 10)
**Type of family** **(couple)**	Nuclear	1		2	
	Joint	11		8	
**Type of marriage** **(couple)**	Arranged	9		5	
	Affair	3		5	
**Age at marriage** **(in years)**	13–19	4	10	3	10
	20–25	3	2	5	-
	>25	5	-	2	-
**Education**	No education	1	-	-	-
	Primary incomplete	-	-	3	1
	Primary complete	3	1	4	1
	Secondary incomplete	5	7	2	8
	Secondary complete	1	-	1	-
	Higher	2	4	-	-
**Occupation**	Skilled jobs	7		5	
	Business	3	-	1	-
	Garment worker	-	-	4	1
	Daily labourer/ Homemaker	2	12	-	8
	Private tuition	-	-	-	1

## Strengths and limitations

The study’s primary strength is its population-based cohort design, which systematically documents life events of newlywed couples concerning sexual and reproductive health and rights (SRHR) in both rural and poor urban settings. The unique selection process for newlyweds and the robust tracking system employed, supported by the Health and Demographic Surveillance System (HDSS) at each site, enhance the internal validity of the study. Utilizing male interviewers for husbands and female interviewers for wives improves data quality and respondent compliance. Moreover, the study’s inclusion of both rural and poor urban areas broadens the comparative scope of various SRHR indicators. To the best of our knowledge, this is the first study to establish a cohort of newlywed couples in Bangladesh.

However, the study has some limitations. Including only newlywed couples from poor urban areas may limit the generalizability of the findings to the broader urban population. Additionally, the necessity of conducting follow-up interviews every four months could introduce recall bias. Furthermore, temporary or long-term spousal separations among migrant workers may affect the reliability of data regarding marital relationships and sexual practices among newlyweds.

## Conclusions

This cohort study is the first longitudinal investigation of its kind in Bangladesh, focusing on the sexual and reproductive health and rights of newlywed couples. It aims to provide insights into how newlywed couples navigate their early years, address their various needs in conjugal life, and cope with the challenges they face. Findings from the study have the potential to highlight critical marital issues that need immediate attention, thereby improving support for recently married couples.

## Supporting information

S1 File IDI guideline. (DOCX)

S2 File Minimal dataset. (XLSX)
