# Correction: Cohort profile: A prospective cohort study on newlywed couples in rural and poor urban Bangladesh

**DOI:** 10.1371/journal.pone.0331301

**Published:** 2025-08-29

**Authors:** Shaki Aktar, Shakil Ahmed, Tanjeena Tahrin Islam, Tarana -E-Ferdous, Meftah Uddin Mahmud, Syed Hassan Imtiaz, Salma Akter, Shathil Miah, Md. Sharful Islam Khan, Ruchira Tabassum Naved, Shams El Arifeen, Quamrun Nahar, Anisuddin Ahmed, S.M. Manzoor Ahmed Hanifi, Fauzia Akhter Huda

The fifth author’s name is incorrect. The correct name is: Meftah Uddin Mahmud. The correct citation is: Aktar S, Ahmed S, Islam TT, -E-Ferdous T, Mahmud MU, Imtiaz SH, et al. (2025) Cohort profile: A prospective cohort study on newlywed couples in rural and poor urban Bangladesh. PLoS ONE 20(1): e0316230. https://doi.org/10.1371/journal.pone.0316230
