# Supplementary material for: Cohort profile: A prospective cohort study on newlywed couples in rural and poor urban Bangladesh
Source: PLoS One. 2025 Jan 22;20(1):e0316230. doi: 10.1371/journal.pone.0316230 (PMC11753628; doi:10.1371/journal.pone.0316230)
Supplement: S1 File — (DOCX) [file pone.0316230.s001.docx]

**ANNEX 5: IDI FACESHEET FOR NEWLYWED SUBJECTS**

**Protocol Title:** Understanding the context and needs related to sexual and reproductive health and rights (SRHR) among the newlywed couples in rural and urban Bangladesh

**Organization:** Maternal and Child Health Division, icddr,b

| Place of interview | **……………………………………………………..**  **……………………………………………………..**  **……………………………………………………..** |
| --- | --- |
| Interview start and ending time | **___ ___ : ___ ___**    **___ ___ : ___ ___** |
| Total length of the IDI | **___ : ___ : ___** |

| **SL** | **BROAD THEMES** |
| --- | --- |
|  | **1. Marriage & Overall Health** |
| 1a | Tell us about your marriage. |
| 1b | How is your life going on now? How is your relation with your new family (meaning husband/wife and in-laws)? How is your relation between *your* spouse and *your* family?  What was your expectation before marriage and how is it different in reality? |
| 1c | [If relationships among the spouses and in-laws are *mostly positive*]: How are you and other family members maintaining such good relations?  [If relationships among the spouses and in-laws are *mostly negative*]: How are you dealing/adjusting with your spouse, in-laws and your family members? |
| 1d | What changes do you see in your life compared to your life before marriage? How you are dealing with these changes. What changes has your spouse gone through and how is he/she dealing with the changes? |
| 1e | Was financial transaction involved in your marriage? [if yes], could you please tell us more about it?  [ex-plore on dowry, gift, wedding expenses etc.) |
| 1f | Do you have any income e.g. job, business, income from property or household initiatives, anything else that generates cash or kind that is used in the family)?  Does your income play a role in running the household or spent elsewhere? How does it impact in your relationship with your spouse (and other family members)?  Who took decision on your occupation or lack of occupation? |
| 1g | Who takes decisions on various issues in your family?  Beyond you and your spouse, whose advices are significant in taking a decision? |
| 1h | ***[For females]:*** what is your experience and perception on your menstrual health? Has it changed after your marriage? What is your husband’s involvement addressing your menstrual problems?  ***[For males]:*** what is your experience and perception on your spouse’s menstrual health? What is your involvement addressing her menstrual problems? |
|  | **2. Fertility Preferences** |
| 2a | What is your perception and attitude on having children after marriage? |
| 2b | Are you currently using any family planning methods?  *[If yes]:* How did you decide what type of family planning method to use? Who advises you on this?  *[If not]:* what is your perception on family planning and using family planning methods? |
| 2c | [If using FP methods]: have you or your spouse experienced any adverse effect while using family planning methods? What did you/your spouse do to overcome it? |
|  | **3. Sexual Vulnerability** |
| 3a | Are you and your spouse living together or separately?  [If *currently* living/previously lived *separately*]: Why? Can you please tell us about your life during this time?  [If living seperattely caused problems]: How did you address the problems? |
|  | **4. Care-seeking behaviour** |
| 4a | What health problems did you have before and after your marriage? |
| 4b | How did you seek care for your ailments before your marriage? How do you seek care after you got married? |
|  | **5. Gender-based Violence (GBV)** |
| 5a | After marriage, what behaviors of your spouse did you find likable and dislikeable?  Do you think his/her behaviour changed over time? Do you think it is normal or unexpected? |
| 5b | ***[For females]:*** Does your husband become intimate with you without your consent? If yes: What are your thoughts on it?  ***[For males]:*** Do you become intimate with your wife without her consent? If yes: What are your thoughts on it? |
| 5c | What is your opinion on whether a husband can beat his wife? |
|  | **6. Final Remarks** |
| 6a | Considering all aspects that we discussed, how happy are you in your married life? |
